# Active generation and magnetic actuation of microrobotic swarms in bio-fluids

**DOI:** 10.1038/s41467-019-13576-6

**Published:** 2019-12-10

**Authors:** Jiangfan Yu, Dongdong Jin, Kai-Fung Chan, Qianqian Wang, Ke Yuan, Li Zhang

**Affiliations:** 1 Department of Mechanical and Automation Engineering, The Chinese University of Hong Kong, Shatin, N.T. Hong Kong; 2 Department of Biomedical Engineering, The Chinese University of Hong Kong, Shatin, N.T. Hong Kong; 3 Chow Yuk Ho Technology Centre for Innovative Medicine, The Chinese University of Hong Kong, Shatin, N.T. Hong Kong; 4 T-Stone Robotics Institute, The Chinese University of Hong Kong, Shatin, N.T. Hong Kong; 5 Shenzhen Research Institute, The Chinese University of Hong Kong, Shenzhen, China

**Keywords:** Magnetic properties and materials, Colloids, Self-assembly

## Abstract

In nature, various types of animals will form self-organised large-scale structures. Through designing wireless actuation methods, microrobots can emulate natural swarm behaviours, which have drawn extensive attention due to their great potential in biomedical applications. However, as the prerequisite for their in-vivo applications, whether microrobotic swarms can take effect in bio-fluids with complex components has yet to be fully investigated. In this work, we first categorise magnetic active swarms into three types, and individually investigate the generation and navigation behaviours of two types of the swarms in bio-fluids. The influences of viscosities, ionic strengths and mesh-like structures are studied. A strategy is then proposed to select the optimised swarms in different fluidic environments based on their physical properties, and the results are further validated in various bio-fluids. Moreover, we also realise the swarm generation and navigation in bovine eyeballs, which also validates the proposed prediction in the ex-vivo environment.

## Abstract (web summary)

Although microrobotic swarms are attractive for biomedical applications, controlling their collective movement in specific bio-fluids is difficult. Here, the authors report navigated locomotion and collective behaviors of magnetic field- and medium-induced micro-robotic swarms in various bio-fluids.

## Introduction

Untethered magnetic micro-/nanorobots have attracted extensive attention due to their great potential for biomedicine at small scales^1–5^. However, due to the small sizes and volumes, the loading capability for drugs of a single microrobot may meet a critical limitation. Meanwhile, the realtime in vivo imaging of a single microrobotic agent is also very challenging. To tackle these challenges, swarm actuation and control strategies are required to be investigated^1,6–8^. Moreover, compared with independent microrobots, the swarms are capable of maintaining controllable patterns during locomotion with a proper global input. By tuning the actuation parameters, microrobotic swarms can perform pattern reconfigurations, which makes them more adaptive to fit inside complex environments for a high rate to access the target. Previously, it has been reported that collective behaviours can be triggered by multiple external stimuli, e.g. magnetic fields^9^, light^10,11^, ultrasound^12^, electrostatic fields^13–16^, and chemicals^17^. By developing proper strategies, dynamic swarms can be actuated with controlled patterns^18–22^. Investigating the behaviours of microrobotic swarms in bio-fluids, including navigated locomotion, active drug delivery and sensing, is an important step for their further in vivo applications. For independent swimmers with relatively weak agent–agent interactions, progress has been made. An artificial system of reactive magnetic micropropellers with surface functionalization can move through gastric acid and mucin gels^23,24^. Fluorescent magnetic spore-based microrobots can serve as highly efficient mobile sensing platforms for the detection of toxins in patients’ stool^25^. However, to date, most of the reconfigurable swarms that can serve as robotic end-effectors are investigated in DI water or aqueous chemicals^26^, and the influence of bio-fluids on them still remains unclear.

In this work, we use two typical kinds of magnetic nanoparticle-based swarms, i.e. magnetic field (MF)-induced and medium-induced swarms, to investigate their effectiveness of performing generation and targeted delivery in different bio-fluids. By analysing and summarising the performances of the swarms in fluids with different physical conditions, we propose a strategy to predict the optimised types of swarms that have higher effectiveness in specific bio-fluids. The strategy is firstly validated in four types of bio-fluids, i.e. blood plasma, foetal bovine serum (FBS), hyaluronic acid (HA), and gastric acid. Quantitative analysis on assembly rates, reconfiguration capability and translational velocities of the swarms are also conducted. For further in vitro validation, we investigate the swarming performances in whole blood and bovine vitreous humour, respectively. Finally, an ex vivo demonstration of applying the optimised swarm in a bovine eyeball is presented. The validation results are consistent with the predictions indicated by our proposed strategy.

## Results

### Magnetic active microrobotic swarm library

**Fig. 1:**
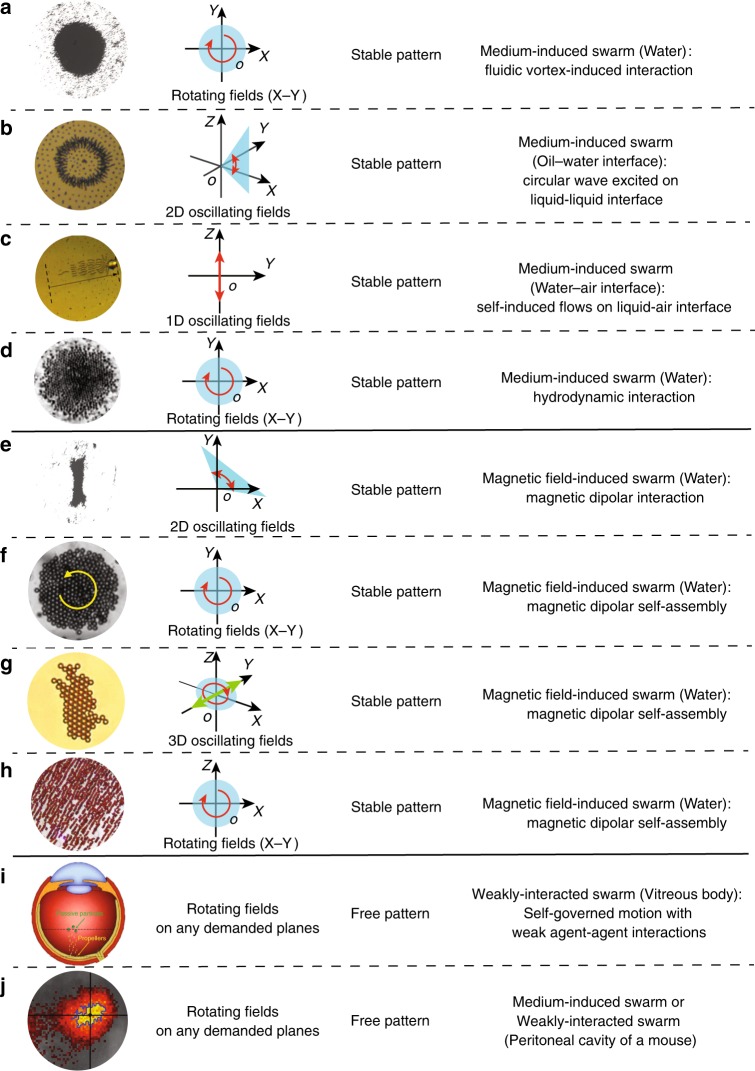
A library of magnetic active swarms. **a** Paramagnetic nanoparticle vortex-like swarm can be generated using rotating magnetic fields, and the merging of fluidic vortices is the main reason for the generation^20^. **b** Magnetic microparticles arrange into an aster on liquid–liquid interface, which is mainly due to the self-induced circular standing wave^19^. **c** A snake-like pattern is formed by magnetic microparticles on liquid–air interface actuated by an oscillating field^28^. **d** A circular pattern formed by rotating helical swimmers^29^. **e** Paramagnetic nanoparticles form reconfigurable ribbon-like swarms actuated by oscillating magnetic fields^21^. **f** By combining magnetic fields and ultrasound fields, superparamagnetic particles can assemble with each other due to dipole-dipole interaction under a rotating magnetic field^30^. **g** Manipulation and propulsion of magnetic colloidal carpets realised by applying conical oscillating magnetic fields^31^. **h** Chain-like structure formed by rolling hematite colloids^32^. **i** Actuation of helical nano-swimmers in vitreous humour^33^. **j** A swarm of artificial bacterial flagella can be navigated in vivo, in the peritoneal cavity of a mouse by applying rotating magnetic fields^7^. The experimental planes of **a**–**h** are X–Y planes. For **i**, **j** the swimmers perform 3D locomotion, and the rotating plane of the magnetic field changes with the desired moving direction.

To date, various kinds of swarming behaviours in colloidal systems have been reported, which can be divided into two major classes: equilibrium and active. While the mechanisms of equilibrium collective behaviour are widely investigated and are relatively well understood, the investigation of active collective behaviour still meets formidable challenges^27^. Moreover, since the active swarms have great potential to be navigated in hard-to-reach location inside human body, they are promising to be employed as microrobots for minimally invasive intervention. Herein, we categorise the out-of-equilibrium microrobotic swarms actuated by magnetic fields into three types by major triggering interactions, as shown in Fig. 1, and the corresponding actuation magnetic fields are also listed. Fluidic interaction can play an important role for the generation of many collectives, such as the vortex-like paramagnetic particle-based swarm (Fig. 1a)^20^, the reconfigurable magnetic aster on a liquid–liquid interface (Fig. 1b)^19^, the snake-like swimmer on a liquid–air interface (Fig. 1c)^28^, and the circular pattern formed by rotating helical swimmers (Fig. 1d)^29^. These swarms are generated mainly due to the interaction exerted through the medium, e.g. hydrodynamic flow and interface, which are defined as medium-induced swarms. In contrast, field-induced swarms are generated from direct agent–agent interactions induced by applied external fields, e.g. magnetic fields, ultrasound and light. The related locomotion of the agents is realised by field-induced forces, and fluidic interaction plays a minor role during the swarm generation. By applying dynamic magnetic fields, ultra-extensible ribbon-like swarms (Fig. 1e)^21^, neutrophil-inspired superparamagnetic particle swarms (Fig. 1f)^30^, magnetic microparticle carpets (Fig. 1g)^31^, and rolling hematite chain-like structures (Fig. 1h)^32^ are generated from colloidal systems. In addition, when the agent–agent interactions are not sufficiently strong to influence the motion of the agents inside the swarm, and they move almost independently, this type of swarm is categorised as a weakly-interacted swarm. Multiple helical nano-swimmers with surface functionalisations are able to be actuated to penetrate vitreous body (Fig. 1i)^33^. A swarm of artificial bacterial flagella with near-infrared probes have been magnetically navigated in vivo, in the peritoneal cavity of a mouse, to demonstrate the possibility of active drug delivery (Fig. 1j)^7^. The agents in the swarm may induce strong flow to influence each other, and in this case, the swarm is categorised into a medium-induced swarm; if the agents move almost independently, it is a weakly-interacted swarm.

**Fig. 2:**
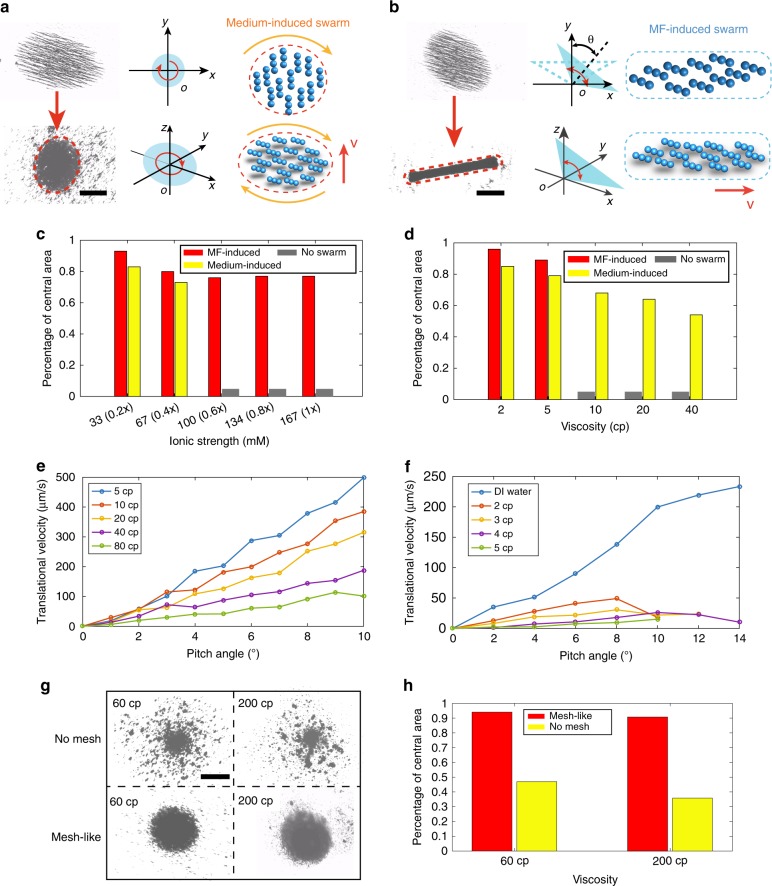
Generation and locomotion of the swarms in ionic and viscous fluids. **a** Schematics and an experimental image of the medium-induced swarms. The actuation fields are indicated by the blue planes. **b** Schematics and an experimental image of the MF-induced swarms. Oscillating magnetic fields are used to trigger the swarm behaviour, and the fields are shown by the blue planes. When the direction of the oscillating field changes, the angle between the centre line and *y*-axis is labelled using $\theta$. The red dashed ellipses indicate the central areas of the swarms. **c**, **d** The percentages of the central areas of the swarms in fluid with different ionic strengths and viscosities, respectively. The ionic strengths of the fluids are normalised using PBS solution, and the ionic strength in PBS solution is 1.0$\times$. **e**, **f** The translational velocities of the medium-induced and MF-induced swarms in fluids with different viscosities, respectively. **g** The experimental results of the medium-induced swarms generated in fluids with and without mesh-like structures. The viscosities of the fluids are kept the same, i.e. 60 and 200 cp, respectively. The shear rate is 5 s${}^{-1}$. **h** The comparisons between the central percentages of the medium-induced swarms generated in fluids with and without mesh-like structures. The scale bars indicate 500 μm.

Restricting most of the agents inside a stable swarming pattern with synchronised motion is critical for targeted delivery in environments with complex branches, which guarantees a high efficiency and a high access rate compared with the deliveries using the weakly-interacted swarms. Therefore, in this work, we mainly focus on the characteristics of medium-induced and MF-induced swarms in bio-fluids. Two swarms are chosen as representative examples, i.e. vortex-like circular swarms (Fig. 1a) as the medium-induced swarm, and ribbon-like swarms (Fig. 1e) as the MF-induced swarm. Regarding to a vortex-like swarm, interaction via fluidic interactions is the main reason for its generation, while for a ribbon-like swarm, the critical actuation interaction that leads to its generation is agent–agent magnetic attractive forces. Detailed explanations about the generation mechanisms of the two swarms are provided in Supplementary Fig. 1, Supplementary Table 1 and Supplementary Note 1. The in-plane rotating magnetic field to generate a vortex-like swarm is shown in Fig. 2a. The rotating magnetic field can be expressed as:

1 $${{\bf{B}}}_{{\rm{Rot}}}={A}_{{\rm{R}}}\left[\sin (2\pi ft){{\bf{B}}}_{{\rm{x}}}+\cos (2\pi ft){{\bf{B}}}_{{\rm{y}}}\right]$$

where ${A}_{{\rm{R}}}$ is the amplitude of the magnetic field, $f$ is the rotation frequency of the field, and $t$ is the time. Alternatively, by applying an oscillating magnetic field, which has a constant component and a sinusoidal component along the direction that is perpendicular to the constant component, a ribbon-like swarm will be formed, as shown in Fig. 2b. The field can be expressed as:

2 $$\begin{array}{lll}{{\bf{B}}}_{{\rm{Osc}}}=\left[{A}_{{\rm{O}}}\sin (2\pi ft)\cos \theta +{C}_{{\rm{O}}}\sin \theta \right]{{\bf{B}}}_{{\rm{x}}}\\ \ \ \ \ \ \ \ \ \ \ + \, \left[{A}_{{\rm{O}}}\sin (2\pi ft)\sin \theta +{C}_{{\rm{O}}}\cos \theta \right]{{\bf{B}}}_{{\rm{y}}},\end{array}$$

3 $$\gamma ={A}_{{\rm{O}}}/{C}_{{\rm{O}}}$$

where ${A}_{{\rm{O}}}$ is the amplitude of sinusoidal signal, ${C}_{{\rm{O}}}$ is the strength of the constant component, and $\theta$ is angle between the constant component and *y*-axis, as demonstrated in Fig. 2b. The amplitude ratio of the two components is represented using $\gamma$. The aspect ratio of the pattern can be reversibly elongated by tuning $\gamma$.

### Swarms in fluids with controlled physical conditions

In order to individually understand how viscosity, ionic strength and polymeric mesh-like structures will influence the nanoparticle swarms, the generation feasibility, reconfigurability and navigated locomotion of the swarms are firstly characterised in artificial fluids with controlled physical properties. We use glycerol–water solution with different concentrations to tune the fluidic viscosity, use phosphate-buffered saline (PBS) to tune the ionic strength, and HA with a concentration gradient to tune the density of the fibrous meshes in fluid. Then, the gathering effect of the swarm in fluids with different ionic strength is investigated, by statistically calculating the area ratio of the swarm central area (the red dashed ellipse and rectangle in Fig. 2a, b, respectively) to the total area of the nanoparticle chains, and the result is shown in Fig. 2c. The data are calculated using Matlab imaging processing programmes. The ionic strengths of the fluids are normalised using PBS solution, which has 1.0$\times$ ionic strength. For the MF-induced swarms, the percentage of the central area decreases from 92 to 75$\%$ with the increase of ionic strength from 0.2$\times$ to 0.4$\times$ PBS solution. With further increase of ionic strength, the gathering effect of the swarm almost maintains in the same range. The percentage of the central area of the medium-induced swarms decreases approximately 12.5$\%$ from 0.2$\times$ to 0.4$\times$ PBS solution. However, if the ionic strength continues to increase, the medium-induced swarm cannot be generated (with fluidic viscosity unchanged). Therefore, medium-induced swarms are more sensitive than MF-induced swarms to ionic strength in fluids during the generation process. Moreover, the ratio of central area of the medium-induced swarms gradually decreases from ~82 to 54% when the viscosity of the fluids increases from 2 to 40 cp, as shown in Fig. 2d. Even though the MF-induced swarms have better gathering effect in 2 and 5 cp, it is unable to be generated in fluids with the viscosities higher than 10 cp, indicating that fluidic viscosity has larger influences on MF-induced swarms.

The translational velocities of the medium-induced and the MF-induced swarms in fluids with multiple viscosities are characterised in Fig. 2e, f. The velocity of the medium-induced swarms becomes lower when the viscosity of fluids is higher, but it can still be up to 100 μm/s when the fluid viscosity is as high as 80 cp. For the MF-induced swarms, the translational velocity encounters a drop when the viscosity of the fluid increases to 2 cp, and with the further increase of the viscosity, the velocity continues to decrease. Therefore, according to the significant velocity decrease, the motion of MF-induced swarms is also more sensitive to fluid viscosity. Moreover, the velocities of the medium-induced and the MF-induced swarms in fluids with multiple ionic strength are presented in Supplementary Figs. 2 and 3, respectively. It can be observed that, ionic strength plays a minor role in affecting the translational velocities of the swarms. The relationships between the aspect ratio of the MF-induced swarm and the input amplitude ratio are shown in Supplementary Figs. 4 and 5 and Supplementary Note 2, respectively.

The influence of the fibrous meshes on the nanoparticle swarm is presented in Fig. 2g, h. Two types of fluids with the viscosity of 60 cp are first prepared, i.e. 80% glycerol–water solution and HA with the concentration of 3 mg/ml. Meanwhile, 89% glycerol–water solution and HA with the concentration of 4.6 mg/mL are also prepared, and their dynamic viscosity is 200 cp (the shear rate is maintained as 5 s^−1^). Glycerol–water solution is a pure fluidic environment without mesh-like structures, while the density of fibrous meshes exist in HA is positively correlated with its concentration. The generation processes of the medium-induced swarms are conducted in these fluids. In high-viscosity fluids with no mesh-like structures, a large part of nanoparticles are left aside during the swarm generation. In HA, however, most of the particles can be gathered into the core part of the swarm, as shown in Fig. 2g. Statistical analysis is also conducted in Fig. 2h. A possible reason is that, during the generation process, the nanoparticles will entangle with the fibrous meshes, and when they are actuated by the rotating magnetic fields, the fibres with the nanoparticles are gathered to form the swarm.

### Selection strategy for optimised swarms

**Fig. 3:**
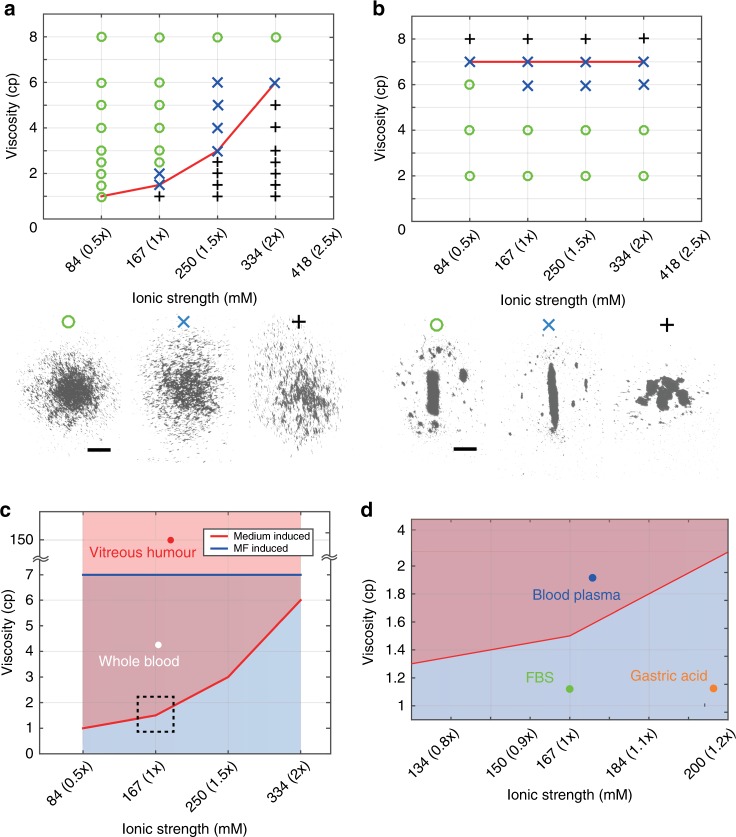
Selection for the optimised swarms in different bio-fluids. **a** Phase diagram for the generation states of the medium-induced swarms. The green circles, blue “×” and black crosses indicate successful generations, generation processes with relatively large loss rate of nanoparticles ($\sim$40%), and failed generations with no swarm patterns, respectively. **b** Phase diagram for the generation states of the MF-induced swarms. Ribbon-like swarms which are capable of performing reversible elongation with a large aspect ratio ($\ge$8) and with a restricted aspect ratio ($\le$3) are indicated by the green circles and blue “$\times$”, respectively. Large clusters will form at the cases labelled by the black crosses. **c** The physical conditions of the fluids that are proper for the generation of the medium-induced and the MF-induced swarms are highlighted by the red and blue regions, respectively. Based on the physical parameters of vitreous humour and whole blood, they are labelled by the red and white spots, respectively. **d** The zoom-in chart of the black dashed square in (**c**). Blood plasma, FBS and gastric acid are labelled using blue, green and orange spots, respectively. By judging the location of the spots, predictions can be made for the optimised swarm type that can be applied. The ionic strengths of the fluids are normalised using PBS solution, and the ionic strength in PBS solution is 1.0$\times$. In **c**, **d** the viscosities are obtained at a shear rate of 10 s${}^{-1}$. The scale bars indicate 500 μm.

Herein, we testify the swarm generation in artificial fluids with different ionic strength and viscosity, from 83.5 to 417.5 mM (0.5$\times$–2.5$\times$ of the ionic strength in PBS) and 2–8 cp, respectively. The generation results of the medium-induced swarms are shown in Fig. 3a. A successful generation is demonstrated by a green circle, and the cases that no swarm can be formed are indicated by the black crosses. When a swarm is generated with a higher particle loss rate (higher than 40$\%$) and its core is relatively loose, the cases are indicated by the blue “$\times$”. A red curve links the boundary cases that a swarm can be generated. With the increase of ionic strength, the swarms can only be generated in fluids with larger viscosities. The medium-induced swarms can be generated in the region higher than the red curve in Fig. 3a. Meanwhile, the generation of the MF-induced swarms in the same series of fluids are presented in Fig. 3b. The link of boundary cases that a ribbon-like swarm can be formed is horizontal, which also indicate that MF-induced swarms are not sensitive to ionic strength in fluids, and the region below the red line are proper conditions for the generation of MF-induced swarms. The fluidic conditions that are proper for the generation of the medium-induced and the MF-induced swarms are demonstrated using the red and blue regions in Fig. 3c, respectively. In the overlap of these two regions, both types of the swarms can be successfully generated. Figure 3d is a zoom-in inset of the black dotted square in Fig. 3c. Plasma, FBS, gastric acid, whole blood and vitreous humour are labelled based on their physical properties as shown in Table 1. The location of each bio-fluid predicts the optimised swarms for the specific fluidic environment. Based on the prediction, both types of the swarms can be generated in whole blood and blood plasma, and only the MF-induced swarms are compatible with the environments of FBS and gastric acid. Moreover, in HA and vitreous humour, where the fluidic viscosity is much higher, medium-induced swarms may be a candidate to be applied.

**Table 1:** Physical parameters of different fluids.

	Viscosity (cp)	Ionic strength (PBS: 1.0$\times$)	Polymeric meshes
DI water	$\sim$0.85	0	No
Plasma	$\sim$1.9	$\sim$180 mM (1.1$\times$)	No
FBS	$\sim$1.1	$\sim$170 mM (1.0$\times$)	No
Gastric acid	$\sim$1.12	$\sim$200 mM (1.2$\times$)	No
HA (5 mg/mL)	$\sim$200	0	Yes
Vitreous humour	$\sim$150	$\sim$170 mM (1.0$\times$)	Yes
Whole blood	4–5	$\sim$180 mM (1.1$\times$)	No

The viscosities are obtained at the shear rate of 10 s${}^{-1}$

### Swarm generation and reconfiguration in bio-fluids

**Fig. 4:**
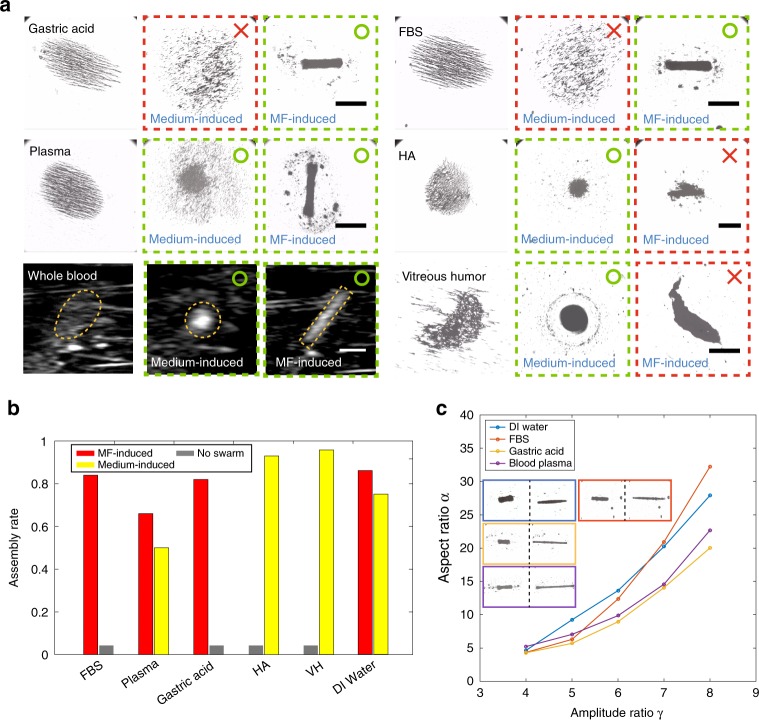
Experimental validations for the prediction. **a** The generation processes of the medium-induced and the MF-induced swarms in gastric acid, FBS, plasma, HA, whole blood and vitreous humour, respectively. Because the red blood cells in whole blood block most of the light from an optical microscopy, ultrasound imaging is applied for the observation. The scales bars represent 800 μm. **b** The assembly rate, which indicates the percentage of swarm central area after the generation process in different bio-fluids. **c** The relationship between the aspect ratio of the MF-induced swarms and the input amplitude ratio. Demonstrations of the elongation of the ribbon-like swarms in DI water, FBS, gastric acid and blood plasma are presented in the insets with blue, red, yellow and purple rectangles, respectively.

The experimental results of swarm generation in various bio-fluids are presented in Fig. 4a and Supplementary Video 1, as validations of Fig. 3c, d. Based on the results, medium-induced swarms can be generated in blood plasma, HA, whole blood and vitreous humour; while MF-induced swarms are effective in gastric acid, FBS, blood plasma and whole blood. It is noted that, in whole blood, ultrasound imaging is applied because optical microscope cannot be used for observation with the red blood cells blocking most of the light. The analytical selection in Fig. 3c, d has a good agreement with the experimental results in Fig. 4a, validating the feasibility of the strategy. Proper magnetic actuation parameters are investigated for swarm generations, as shown in Supplementary Figs. 6–9 and Supplementary Note 3. Percentage of the central area of the swarms generated in different bio-fluids is presented in Fig. 4b. In the cases when both the swarms can be generated, the MF-induced swarms always have better assembly rates in comparison with the medium-induced swarms. For the medium-induced swarms, a significantly high percentage can be observed in HA and vitreous humour, and meanwhile, in plasma, the percentage is relatively lower, as approximately 50%. Due to the relatively large interference from the noise of the ultrasound imaging results, the statistical calculation of the assembly rate is not conducted. The MF-induced swarms are capable of performing reversible pattern elongation and contraction by tuning the input magnetic field. The change in aspect ratios of the MF-induced swarms with input amplitude ratio is shown in Fig. 4c, and the changes of aspect ratio with different input oscillating frequency in respective bio-fluids are presented in Supplementary Fig. 10 and Supplementary Note 4.

### Navigated locomotion in bio-fluids

**Fig. 5:**
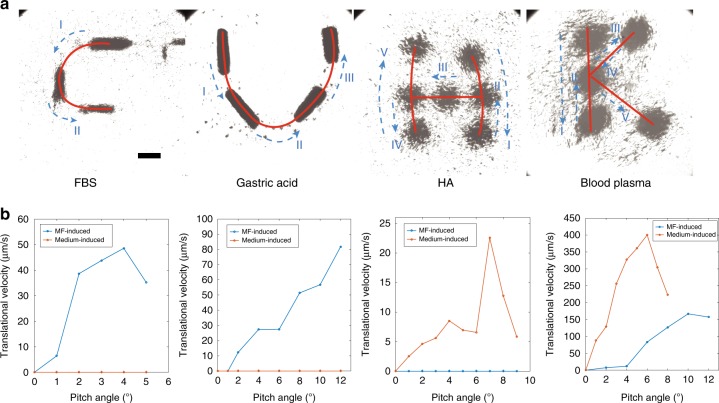
Navigated locomotion of the MF-induced and the medium-induced swarms in different bio-fluids. **a** The locomotion trajectories “CU” are from the MF-induced swarms in FBS and gastric acid, respectively; and the trajectories “HK” are from the medium-induced swarms in HA and blood plasma, respectively. The scale bar indicates 500 μm. **b** The translational velocities of the swarms in different bio-fluids.

The swarms can perform navigated locomotion by applying magnetic fields with pitch angles. The experimental results of navigated locomotion are displayed in Fig. 5a and Supplementary Video 2. The moving trajectories of the MF-induced swarms showing “CU” are performed in FBS and gastric acid, respectively. Meanwhile, the trajectories of the medium-induced swarms showing “HK” are performed in HA and blood plasma, respectively. The relationships between the translational velocity and the applied pitch angles in four different bio-fluids are shown in Fig. 5b. In FBS, the maximum translational velocity of the MF-induced swarms reaches approximately 50 μm/s (pitch angle 4${}^{\circ }$). When the pitch angle becomes higher than 4${}^{\circ }$, the velocity decreases and the swarm pattern gradually becomes unstable. In both FBS and gastric acid, the velocities of the medium-induced swarm are not applicable because they cannot be formed. In HA (5 mg/mL), due to the high viscosity and the fibrous meshes, medium-induced swarm has a relatively low maximum velocity of 23 μm/s. Moreover, these two kinds of swarms are formed well in blood plasma, and the translational velocities are significantly higher than the other three cases.

### Swarms in whole blood

**Fig. 6:**
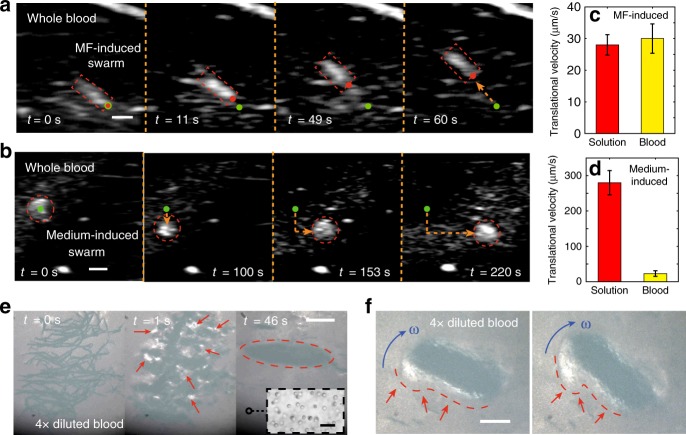
The navigated locomotion of MF-induced and medium-induced swarms in whole blood. **a** The steered locomotion of an MF-induced swarm in whole blood. **b** The navigation of a medium-induced swarm in whole blood. In **a**, **b** the scale bars indicate 1 mm. **c** The comparison between the translational velocities of the MF-induced swarms in glycerol–water solution and in whole blood, with the same viscosity. The applied pitch angle is 10${}^{\circ }$. **d** The comparison between the translational velocities of the medium-induced swarms in glycerol–water solution and in whole blood, with a pitch angle of 6${}^{\circ }$. The error bars in **c**, **d** indicate the standard deviation obtained from three experiments. **e** The generation of an MF-induced swarm in 4$\times$ diluted blood. The red dashed ellipse indicates the formed swarm. **f** The turning motion of an MF-induced swarm in 4$\times$ diluted blood for the investigation of swarm-cell interaction. In **e**, **f**, the scale bars indicate 500 μm. The scale bar in the inset of **e** represents 20 μm. The error bars in **c**, **d** indicate standard deviations obtained from five experiments.

Microrobotic swarms are promising for biomedical applications in vascular system, e.g. dissolving blood clots. Even though the fast blood flow in arteries may significantly influence the generation and locomotion of the swarms, recent clinic approaches, such as catheter and balloon occlusion techniques, make the applications using swarms in blood vessels possible. The demonstration of swarm generation in whole blood has already been shown in Fig. 4a. Herein, the translational locomotion of MF-induced and medium-induced swarms is presented in Fig. 6a, b, respectively. The initial and current locations of the swarms are labelled by the green and red spots, respectively. The navigated locomotion of the swarms can be clearly observed, and their trajectories are labelled by the yellow dashed arrows. In both cases, the swarming patterns keep stable during the locomotion due to the agent–agent interaction. The peak translational velocities of the MF-induced swarm and the medium-induced swarm are approximately 30 μm/s (pitch angle: 10${}^{\circ }$) and 23 μm/s (pitch angle: 6${}^{\circ }$), respectively. As shown in Fig. 6c, d, compared with the cases in glycerol–water solution with the similar viscosities (4–5 cp), MF-induced swarms have a similar peak velocity in whole blood; while the velocity of the medium-induced swarms significantly drops from the glycerol–water solution to whole blood (from 280 to 23 μm/s). As we analysed, ions in fluids exert minor effects on the translational locomotion of both types of the swarms (Supplementary Figs. 2 and 3). Therefore, the red blood cells exert great influences on medium-induced swarms, and their influences on MF-induced swarms are relatively minor.

The phenomenon that the two types of swarms respond differently to the colloidal jamming formed by red blood cells (RBC) can be explained. For the a medium-induced swarm, its stable circular pattern is generated and maintained through the interactions among vortices, which are induced by the rotating particle chains inside it. The overall effect of the rotating flow field tends to attract the nearby RBCs into the centre of the swarm^20^. The blood cells can thus bring significant influence to each vortices, which leads to the drop of its translational velocity. Meanwhile, in order to investigate the interaction between MF-induced swarms and RBCs, demonstrations of swarm generation and turning motion in 4$\times$ diluted blood are presented in Fig. 6e, f and Supplementary Video 3, respectively. Initially, before the magnetic actuation, as shown in Fig. 6e (*t* = 0 s), the contrast of the nanoparticle clusters to the background is relatively low due to the existence of a large number of blood cells. The RBCs are shown in the inset of Fig. 6e. During the swarm generation, the flow fields induced are capable of pushing the blood cells away. With the decrease of cell concentration, several white regions (substrate colour) gradually appear in Fig. 6e (1 s) (a part of the regions is highlighted by red arrows), which indicates that relatively free spaces with few cells are created for the swarm to generate. Moreover, after the ribbon-like swarm is generated, the induced flow still pushes the cells away from its body, as shown by the white regions along the contour of the swarm in Fig. 6f, which is highlighted by the red dashed lines and red arrows. As a result, a ribbon-like swarm can always move in a region with much less blood cells. Meanwhile, because the viscosity of blood is approximately 4–5 cp, and that of plasma (without blood cells) is 1.8–2 cp, by pushing away most blood cells, the swarm actively decreases the viscosity of the blood near its body, which may significantly enhance its moving capability. The experimental results of the navigated locomotion in 4$\times$ diluted blood are shown in Supplementary Fig. 11, Supplementary Note 5 and Supplementary Video 3. It is noted that, the protein corona in blood^34^ will not hinder the swarm behaviour of the nanoparticles, as shown in Supplementary Fig. 12 and Supplementary Note 6.

### Swarms in vitreous humour and bovine eyeballs

**Fig. 7:**
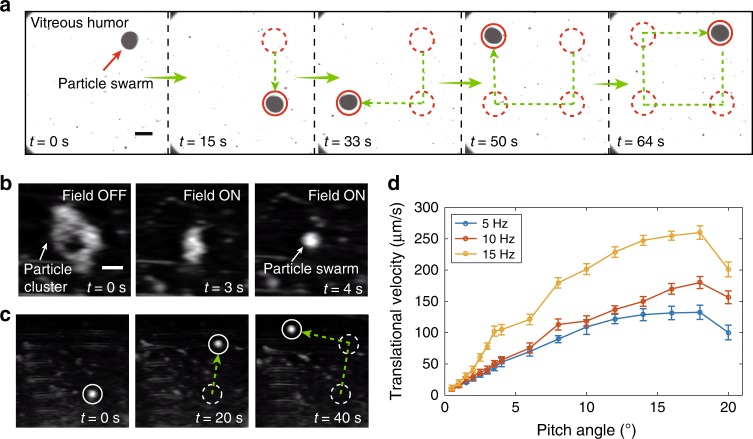
Navigated locomotion of medium-induced swarms in bovine vitreous humour. **a** With optical imaging feedback, the medium-induced swarm is capable of following a rectangular trajectory. **b** The generation process of a medium-induced swarm in vitreous humour. **c** The navigated locomotion process in vitreous humour with ultrasound imaging feedback. The circles indicate the present location of the swarm, the dotted circles indicate the previous location of the swarm, and the green dotted arrows shows the moving trajectories of the swarm. **d** The translational velocity of the swarms formed by nanoparticles after hydrophobic surface treatment. The scale bars indicate 600 μm. The error bars in **d** indicate standard deviations obtained from three experiments.

In order to enhance the moving capability of the swarm, surface functionalization is firstly performed to the nanoparticles to make them hydrophobic. The treatment steps and the preparation of vitreous humour are described in the “Methods” section. The navigated locomotion of the medium-based swarm in bovine vitreous humour has been demonstrated in Fig. 7a. The swarm is capable of performing navigated locomotion to follow a rectangular trajectory with good motion controllability. A rotating magnetic field with a strength of 7  mT and rotating frequency of 18 Hz is applied. During the locomotion, the fast-rotating swarm can still keep its pattern intact, and meanwhile, very few particles will be left along the trajectory. This is a very important advantage in applications like eye therapies, because even a very low portion of nanoparticles left inside the eyes will significantly influence the vision. Furthermore, from the Supplementary Video 4, the motion of the swarm is also different from that in other bio-fluids we used. When the swarm moves, it exerts fluidic attractive forces on other small nanoparticle clusters and impurities in vitreous humour (the small black dots in Fig. 7a). When the impurities are attracted close to the swarm, due to the dense mesh, they exert relatively large influence to the swarm, which leads to slight vibration of the swarm during the locomotion. It is noted that, the vibration only has minor effect on the navigation precision of the swarm in a short time period, the overall direction controllability still remains unaffected. In the demonstration, the average velocity of the swarm is ~200 μm/s. Previously, Wu et al. reported that slippery helical nanoswimmers (3 μm long with a diameter of 300 nm) are capable of swimming inside vitreous humour by penetrating through the mesh pores^33^. The propulsion force generated by the nanoswimmers can be estimated at the pico-newton level^35,36^, and in this case, the strength of the collagen fibres in vitreous humour is overwhelming. In contrast, the vortex-like swarm in our work is at the millimetre/sub-millimetre scale. By applying tracing particles around the swarm, and observing the motion of them, the propulsion force generated by the swarm is estimated to be 1 $\sim$ 8 μN by combining Stokes’ law and Weissenberg number^37^, which is comparable to the data given for the magnetic agents with a similar size^38^. As a result, the strong fluidic forces generated by the swarm could be capable of pushing and twisting the meshes in vitreous humour^38^, which is the possible mechanism to explain the translational locomotion of the swarm. From the results as shown in Supplementary Figs. 13 and 14 and Supplementary Note 7, it can be concluded that the navigated locomotion of the swarm is not dependent to the partial dilution or the overall dilution of the vitreous humour due to the injected suspension. The physical property (i.e. viscoelasticity) of the vitreous before and after the actuation study of the swarm is also characterised, as shown in Supplementary Fig. 14, and the results are in the same range with minor deviations. Therefore, the injection of particle suspension and swarm actuation will bring negligible effects to the vitreous humour.

Moreover, to realise in vivo therapies in eyes, ultrasound imaging is one of the major methods to obtain an optimal broad view when there is a dense cataract or vitreous haemorrhage in the eyes^39^. We investigate the swarm generation and navigated locomotion with feedback using ultrasound imaging, as shown in Fig. 7b, c (Supplementary Video 5), respectively. At *t* = 0 s, the spread nanoparticle clusters are shown by the white irregular pattern. After a rotating magnetic field is applied, the pattern shrinks immediately and finally forms a circular pattern (at *t* = 4 s). After the swarm is generated, the imaging contrast is improved compared with the initial stage, which provides convenience for the following tracking of navigated locomotion. As shown in Fig. 7c, the swarm is navigated to follow a right-angled path. Finally, the translational velocities of the swarms formed by hydrophobic magnetite nanoparticles are shown in Fig. 7d. The maximum translational velocity that can be reached by the swarm is over 250 μm/s (15 Hz).

**Fig. 8:**
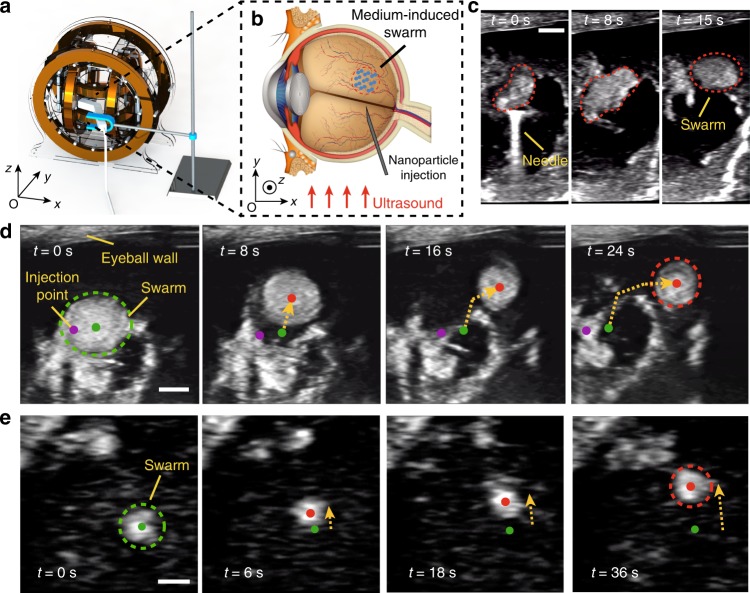
Ex vivo validations of the generation and actuation of medium-induced swarms in bovine eyeballs. **a** The schematic demonstration of our experimental setup. An ultrasound transducer is integrated onto the Helmholtz electromagnetic coils. A bovine eyeball is placed in the workspace. **b** A schematic illustrating the navigated locomotion of a medium-induced swarm in vitreous. **c** The generation process of a swarm after the injection of nanoparticle suspension using a syringe. The pattern of the nanoparticles is inclosed by red dashed curves. The scale bars indicate 1 mm. **d** The targeted locomotion of a large-size swarm. The scale bars indicate 2 mm. **e** The targeted locomotion of a relatively small-size swarm. The scale bars indicate 800 μm. The original pattern and the centre are indicated using a green dashed ellipse and a green spot, respectively. The injection point is indicated by purple spots. The current centre of the swarm is presented by red spots, and the trajectories of the locomotion are indicated using yellow dashed arrows.

In order to further validate the feasibility of our selection approach in an ex vivo environment, we conduct swarming generation and navigated locomotion in bovine eyeballs. A Helmholtz electromagnetic coil setup integrated with an ultrasound transducer is used for the experiments, as shown in Fig. 8a and Supplementary Fig. 15. The setup is also used for the aforementioned experimental results with ultrasound feedback. A bovine eyeball is placed in the workspace of the setup, and concentrated particle suspension (6  mg/mL) is injected into the eyeball using a syringe. Then, a rotating magnetic field is applied to trigger the swarm behaviour of the nanoparticles, as schematically illustrated in Fig. 8b. The generation process is demonstrated in Fig. 8c and Supplementary Video 6. The syringe needle for injection can be clearly found, which is helpful to locate the region of the injected nanoparticles for further observation. After the injection, the nanoparticles initially form irregular patterns, and then, a medium-induced swarm with an elliptical pattern is gradually formed at 15 s. The coverage patterns of the nanoparticles are shown by the red dashed curves in Fig. 8c. The successful generation process has good agreement with our prediction (Fig. 3c, d) and the preliminary experiments in VH (Fig. 4a).

Furthermore, the locomotion capability of formed swarms is testified in the bovine eyeball, and the results are shown in Fig. 8d, e. The injection point of the nanoparticle suspension is indicated by the purple spots in Fig. 8d. It is noted that the volume of the nanoparticle suspension injected into the bovine eyeball is approximately 0.2 mL, which is a small value compared with the volume of the vitreous humour ($\sim$5 mL). Meanwhile, the locomotion distance of the swarm is far larger than the original injection range, and as a result, the translational motion of the swarm is not dependent on the partial dilution of the vitreous humour (Supplementary Fig. 13). The green and red spots indicate the original and current locations of the swarms, respectively. A large-size swarm with a radius of ~2.2 mm is generated as shown in Fig. 8d. In this case, the applied rotating magnetic field has a magnetic strength of 9 mT, and a rotating frequency of 12 Hz. By applying 5${}^{\circ }$ pitch angle with the rotating field, the swarm can be actuated and navigated, as shown in Supplementary Video 7. Herein, the swarm is steered to avoid the collision with the eyeball wall, which is labelled in Fig. 8d. The moving trajectory is shown by the yellow dashed curves. The total translational locomotion of the swarm is over 6 mm within 24 s which indicates that the swarms are promising for performing efficient targeted drug delivery inside eyeballs. In order to demonstrate the scalability of the swarming actuation strategy, relatively small swarms are investigated, and the experimental results are shown in Supplementary Videos 8 and 9. In Fig. 8e, a swarm with a radius of 500 μm is actuated, and it can also be steered with external magnetic fields, towards the direction indicated by the yellow dashed arrows. In this case, the average velocity of the smaller swarm is ~56 μm/s. Furthermore, to validate the wide application in complex bio-fluids, we also realise the actuation of swarms on a mucosa sample (i.e. intestinal tract) with a mucus layer, and the results are shown in Supplementary Fig. 16 and Supplementary Note 8. Cytotoxicity tests of the nanoparticles are conducted, as shown in Supplementary Fig. 17 and Supplementary Note 9, and the results show that the nanoparticles have low cytotoxicity to both normal cells (3T3 cells) and cancer cells (HeLa cells and HepG2 cells).

## Discussion

In this work, we investigate the generation and remote actuation of the medium-induced and the MF-induced swarms in bio-fluids. By analysing and summarising the performances of the swarms in fluids with different physical conditions, we propose a strategy for selecting the optimised swarms in specific bio-fluids. Besides gastric acid, FBS, blood plasma and HA, two types of bio-fluids that are directly extracted from living bodies are used for validation, i.e. whole blood and vitreous humour. As a result, we validate that MF-induced swarms are not sensitive to ionic strength, and it can be generated in fluids with relatively lower viscosities. Other field-induced swarms actuated by different stimulus may also be sensitive to fluidic viscosity, e.g. the increased viscosity may hinder the formation of the linear collective array of metallic rods actuated by ultrasound^40^. In contrast, medium-induced swarms have the potential to be applied in fluids with higher viscosities and fibrous meshes. The experimental validations have good agreements with the prediction. Finally, we demonstrate the ex vivo generation of medium-induced swarms and their targeted deliveries in a bovine eyeball. This work sheds light on the fundamental understanding of microrobotic swarms, and it is an important step further towards the in vivo targeted delivery.

## Methods

### Synthesis of hydrophobic nanoparticles

In our experiments, magnetite nanoparticles with a diameter of 100 nm are prepared using solvothermal method, which has been previously reported^41^. Typically, 50 mg Fe${}_{3}$O${}_{4}$ nanoparticles were first mixed with 80 mL ethanol, 20 mL DI water and 1 mL ammonium hydroxide aqueous solution (28%), followed by vigorous mechanical stirring and sonication for 15   min. Next, 0.15 mL tetraethyl orthosilicate was added dropwise and the mixture was allowed to react for 6 h under room temperature. The nanoparticles were then collected by a permanent magnet, washed with ethanol for 5 times and dispersed in 100 mL ethanol. Afterward, 100 μL trichloro (1H,1H,2H,2H-perfluoroctyl) silane was injected into the nanoparticle solution, which was further mechanically stirred for 24 h. Finally, the product was collected, washed by ethanol and water for 5 times in sequence, and dispersed in DI water with a concentration of 4 mg/mL. Before magnetic actuation, the synthesised nanoparticles are subjected to an ultrasonic bath for 5 min for a better suspension state.

### Preparation of vitreous and magnetic actuation setup

Bovine eyes were obtained from a local slaughter house, and they are stored on ice (0${}^{\circ }$–4${}^{\circ }$) during transportation. The post-mortem storing time of the eyeballs is less than 15 h before the experiments. With this storing condition, the mechanical properties of vitreous humour can basically keep the same with those of fresh samples within this period^42^. The VH samples for the in vitro investigations are extracted from the bovine vitreous using a syringe, and are used within 1 h. The magnetic actuation and control experiments are conducted in a 3-axis Helmholtz electromagnetic coil setup (Supplementary Fig. 1e). The control signals are generated by a PC, and then the current is applied into the coils to generate magnetic fields in the working space. We are able to use the setup to generate rotating and oscillating magnetic fields with specific requirements, by inputting mathematical expressions into the control programme. For in vitro investigations, one drop of the nanoparticle solution (3 μL, 3 mg/mL) is added into a tank, which is filled with bio-fluids ($\sim$0.7 mL). The diffused particles are further gathered using a magnet field gradient. In order to create a uniform distribution of the nanoparticle chains, a reported dynamic magnetic field with a frequency of 20 Hz is applied to disassemble the particle clusters while spread them^43^. After the disassembly process, the nanoparticles are ready for further magnetic actuation processes.

### Ex vivo experiments in a bovine eyeball

The eyeball is firstly placed at the centre of the working space of the Helmholtz coil setup with the its pupil sideways (Fig. 8b). The nanoparticles are suspended in PBS buffer with a concentration of 6 mg/ml, and the 200 μL of the suspension is injected into the eyeball using a syringe. The injection point is at the side of the eyeball (Fig. 8b). After the injection, the rotating magnetic field is turned on immediately in order to generate a vortex-like swarm, with a field strength of 10 mT and a frequency of 8 Hz. The applied pitch angle for actuating the swarm to make locomotion is 5${}^{\circ }$. During the actuation, an ultrasound system (Terason t3200, Teratech Corporation, USA) is used for the observation, and the ultrasound transducer (16HL7, Teratech Corporation, USA) is fixed to just contact the outer eyeball wall during the process. No significant force shall be generated due to the eye-transducer contact, in case of change in the pressure inside the eyeball.

## Supplementary information

Supplementary Information

Description of Additional Supplementary Files

Supplementary Movie 1

Supplementary Movie 2

Supplementary Movie 3

Supplementary Movie 4

Supplementary Movie 5

Supplementary Movie 6

Supplementary Movie 7

Supplementary Movie 8

Supplementary Movie 9

## Data Availability

All the relevant data used to prepare this paper and the Supplementary Information is available upon request.

## Code availability

All the relevant code used to generate the results in this paper and the Supplementary Information is available upon request.
