# Supplementary material for: Active generation and magnetic actuation of microrobotic swarms in bio-fluids
Source: Nat Commun. 2019 Dec 10;10:5631. doi: 10.1038/s41467-019-13576-6 (PMC6904566; doi:10.1038/s41467-019-13576-6)
Supplement: Supplementary file 1 — Supplementary Information [file 41467_2019_13576_MOESM1_ESM.pdf]

Supplementary Information

Active Generation and Magnetic Actuation of Microrobotic Swarms in Bio-fluids

Jiangfan Yu[1,2], Dongdong Jin[1,3], Kai-Fung Chan[3], Qianqian Wang[2], Ke Yuan[3], and Li Zhang[2,3,4,5,6*]

[1] The authors contributed equally to this work.

[2] Department of Mechanical and Automation Engineering, The Chinese University of Hong Kong, Shatin, N.T., Hong Kong SAR, China.

[3] Department of Biomedical Engineering, The Chinese University of Hong Kong, Shatin, N.T., Hong Kong SAR, China.

[4] Chow Yuk Ho Technology Centre for Innovative Medicine, The Chinese University of Hong Kong, Shatin, N.T., Hong Kong, China.

[5] T-Stone Robotics Institute, The Chinese University of Hong Kong, Shatin, N.T., Hong Kong, China.

[6] Shenzhen Research Institute, The Chinese University of Hong Kong (Shenzhen), China.

*To whom correspondence should be addressed. E-mail: lizhang@mae.cuhk.edu.hk

Supplementary Figures

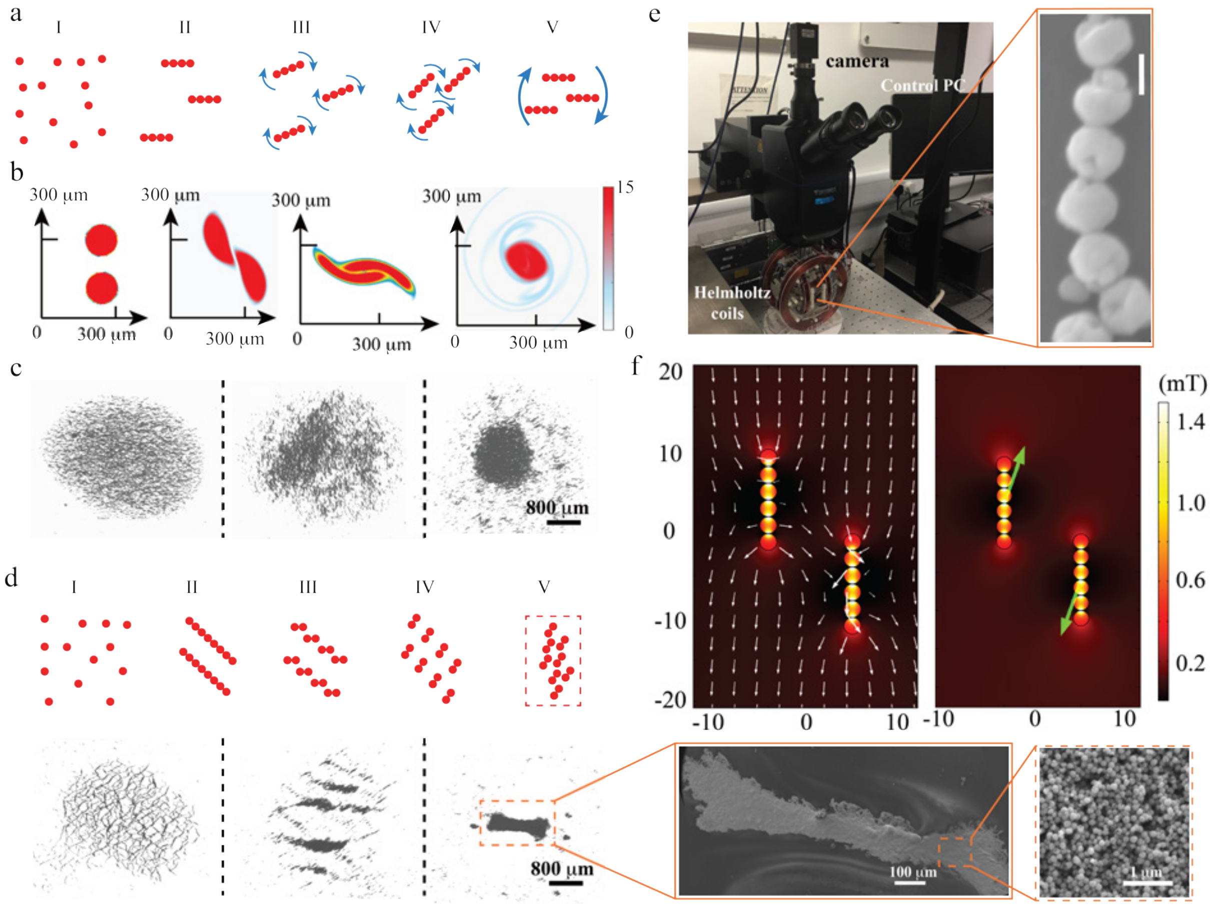

Supplementary Figure 1: Generation mechanisms of the swarms. (a) The schematic demonstration of the generation process of a fluidic-induced swarm. (b) The simulation results of the merging process of two identical vortices induced by rotating nanoparticle chains. The colour map shows the vorticity field. The simulation is conducted through Matlab from analytical equations. (c) The experimental results of the generation process of a fluidic-induced swarm in DI water. (d) The schematic demonstration and experimental results (in DI water) of the generation of a MF-induced swarm. Two SEM images showing the details of the swarm are shown in the inset highlighted by the orange rectangle. (e) The magnetic actuation setup in this work. A SEM image showing the magnetic nanoparticles is presented in the inset. The scale bar is 100 nm. (f) The simulation results of the magnetic interaction between two paramagnetic nanoparticle chains. The simulation is conducted using Comsol.

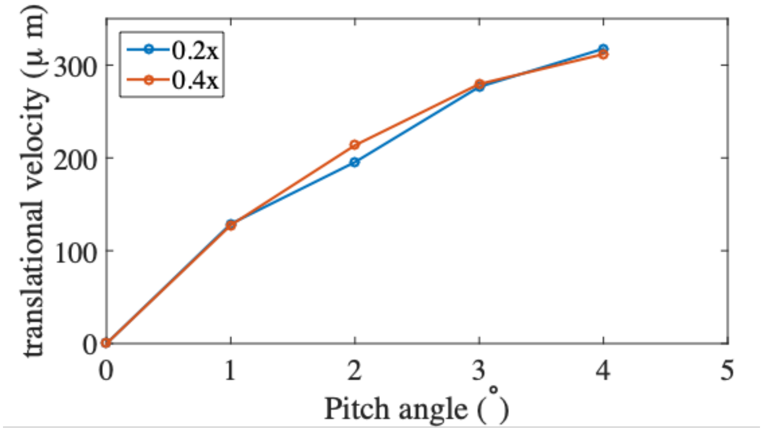

Supplementary Figure 2: The translational velocities of medium-induced swarms in ionic fluids. The ionic strength in PBS is 1×.

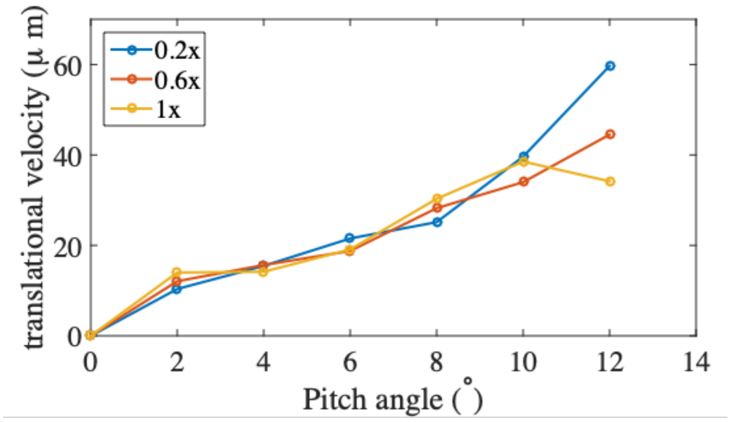

Supplementary Figure 3: The translational velocities of MF-induced swarms in ionic fluids. The ionic strength in PBS is 1×.

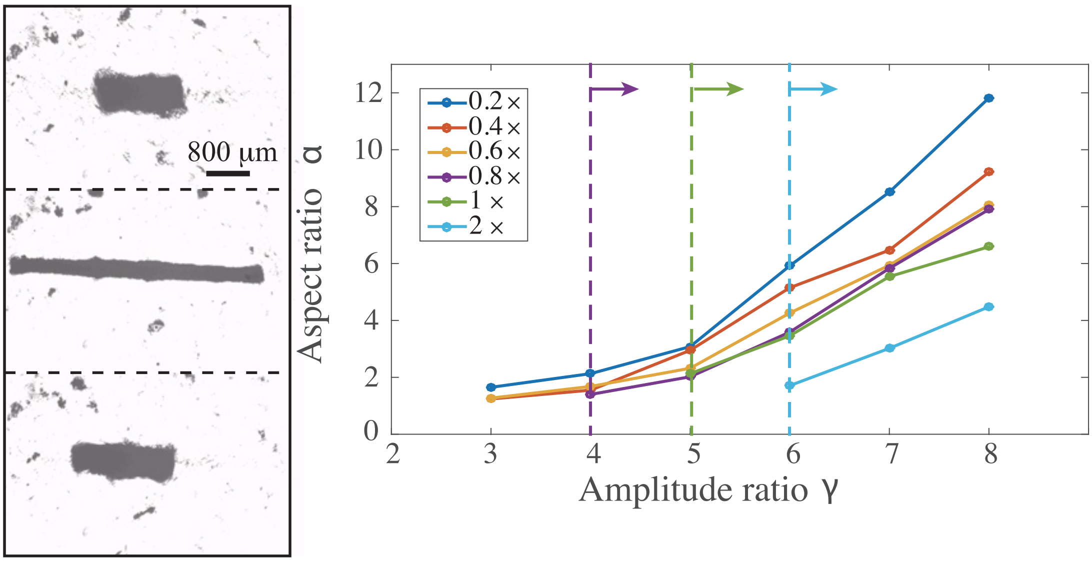

Supplementary Figure 4: The change of the α with γ in fluids with different ionic strengths. The reversible elongation of an MF-induced swarm is demonstrated in the black rectangle.

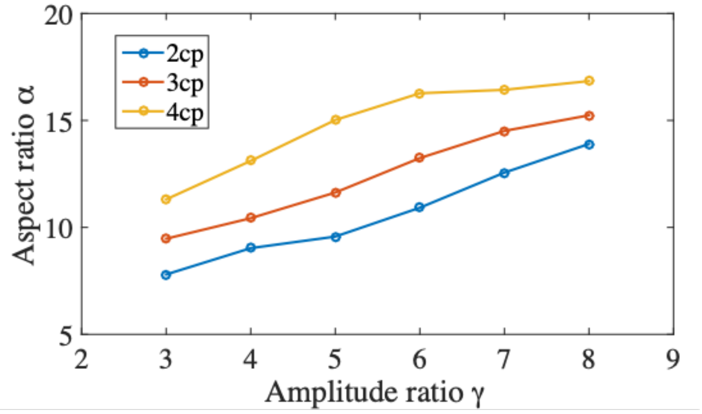

Supplementary Figure 5: The change of α of an MF-induced swarm with γ in viscous fluids.

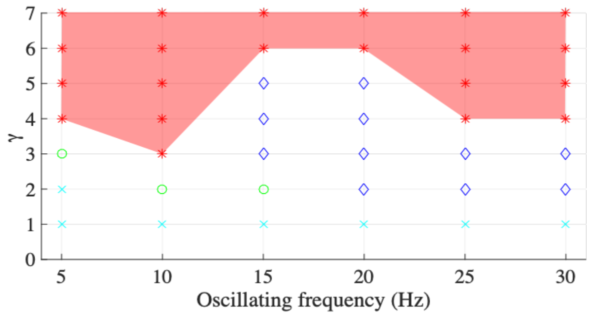

Supplementary Figure 6: The successful generation conditions of MF-induced swarms in gastric acid.

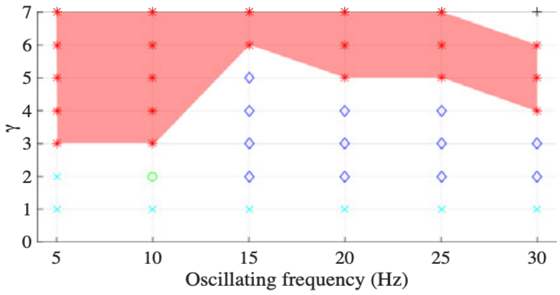

Supplementary Figure 7: The successful generation conditions of MF-induced swarms in plasma.

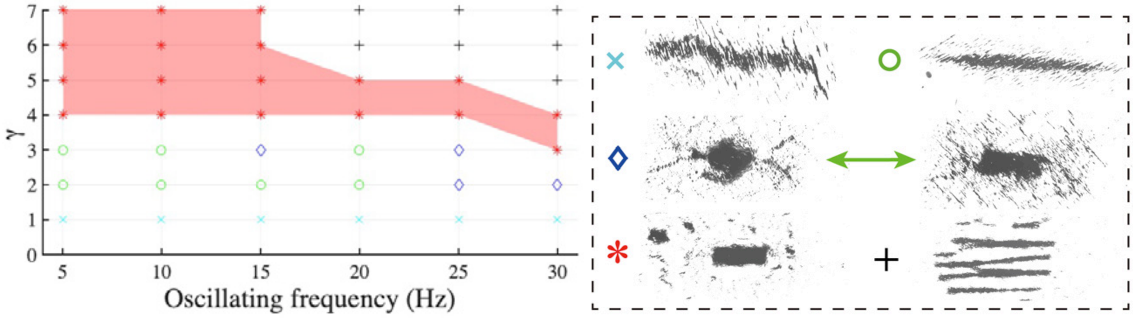

Supplementary Figure 8: The successful generation conditions of MF-induced swarms in FBS.

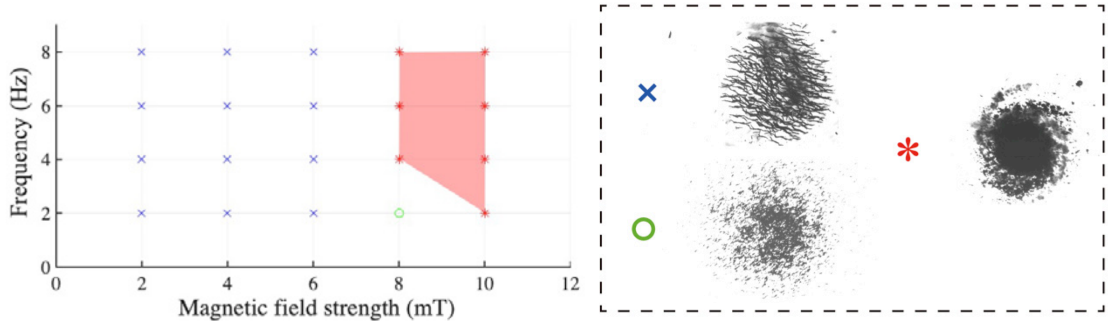

Supplementary Figure 9: The successful generation conditions of medium-induced swarms in HA.

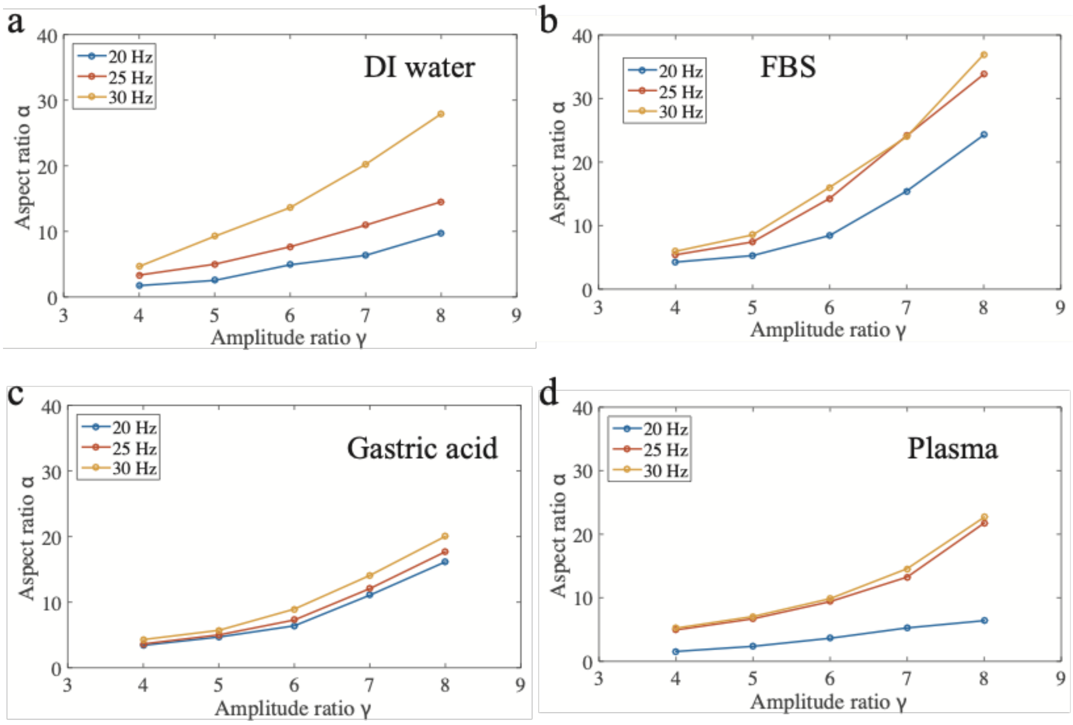

Supplementary Figure 10: The relationships between the α of MF-induced swarms and γ in bio-fluids. The features are testified in (a) DI water, (b) FBS, (c) gastric acid and (d) plasma, respectively.

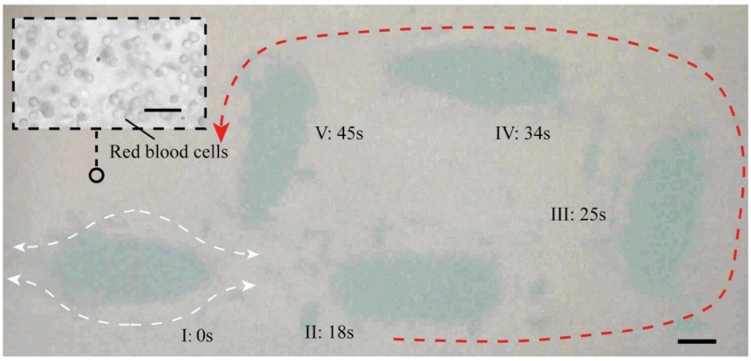

Supplementary Figure 11: The navigated locomotion of an MF-induced swarm in 4× diluted blood. The trajectories of the blood cells at the two tips of the swarm is schematically demonstrated using white dashed lines. The scale bar indicates 200 μm. The scale bar in the inset indicates 20 μm.

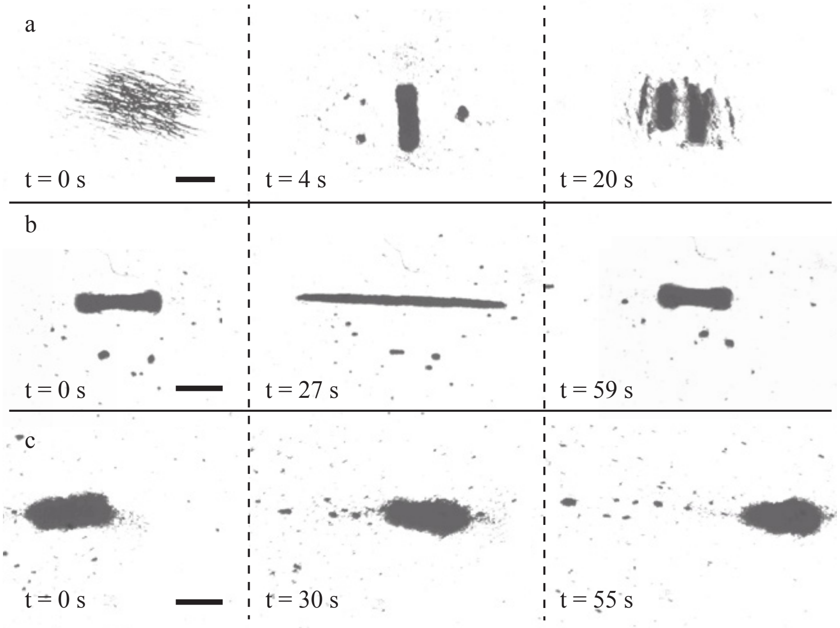

Supplementary Figure 12: The ribbon-like swarm formed by corona-coated nanoparticles in blood plasma. The scale bars indicate 500 μm.

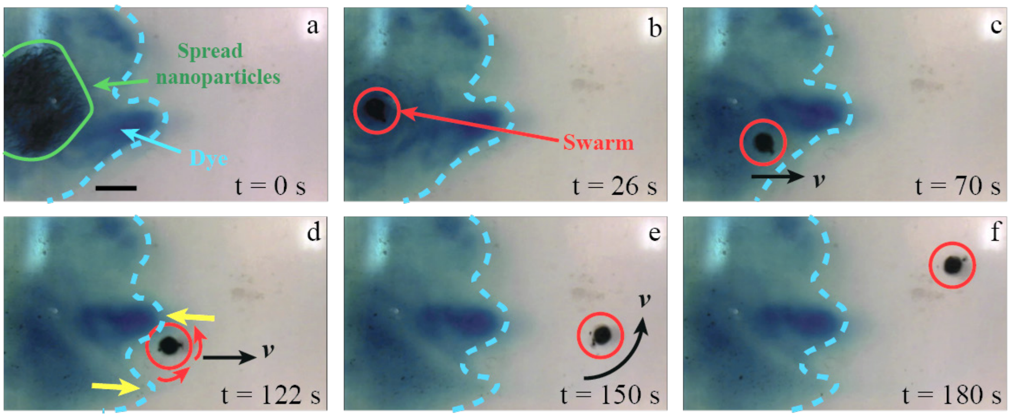

Supplementary Figure 13: The locomotion capability of the swarm inside and outside the region of dilution. The nanoparticles are suspended in blue dye, and the suspension is injected into the vitreous humor. (a) The spread nanoparticles, which is indicate by the green contour. (b) After the rotating field is applied, the swarm is generated, which is highlighted by the red circle. (c) - (f) The locomotion of the swarm. The direction of the translational velocity of the swarm is shown by the black arrows, the rotation of the swarm is indicated by the red arrows, and the contour of the dyed region is represented using blue dashed curves. The yellow arrows show the major change of the dyed contour. The scale bar indicates 2 mm.

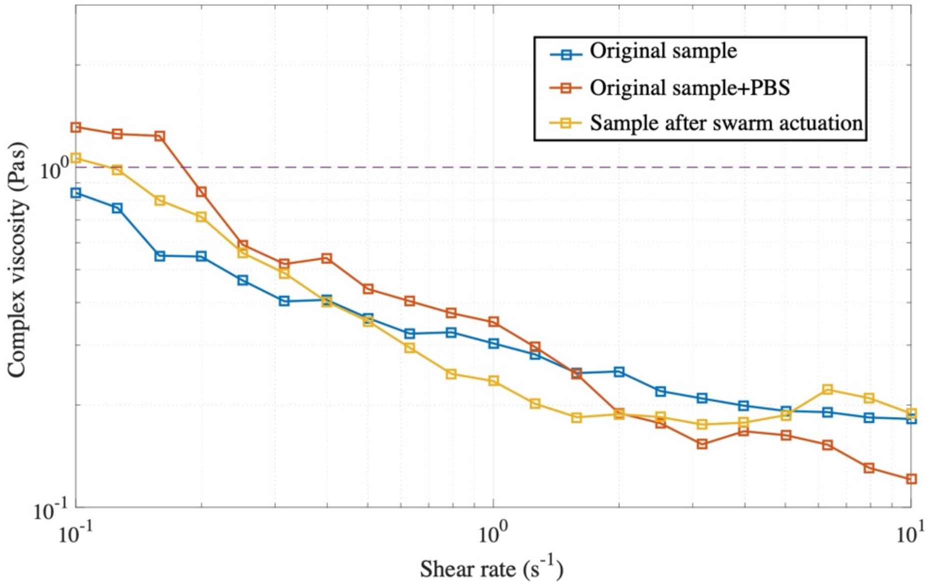

Supplementary Figure 14: The viscoelasticity of vitreous humor with different conditions. Fresh vitreous humor, the sample of mixed vitreous with PBS solution, and the sample after the actuation experiments are applied. The proportion of vitreous humor and PBS solution is kept the same with the ex-vivo experiments in bovine eyes (Figure 7). A swarm is actuated inside the vitreous for approximately 2 min.

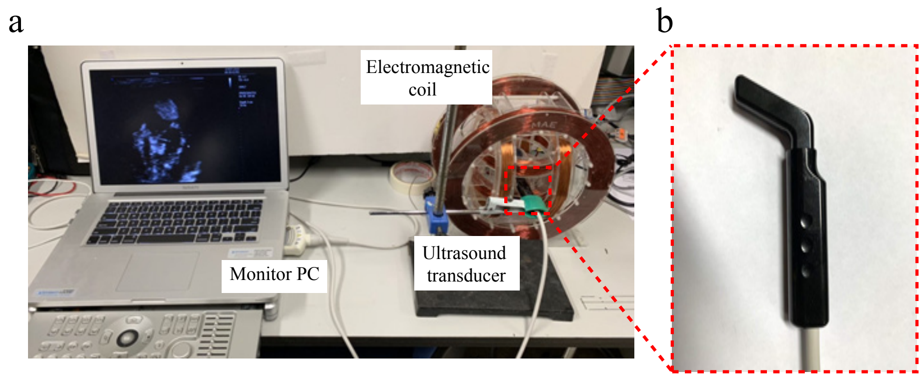

Supplementary Figure 15: The setup for the experiments in bovine eyeballs. (A) The Helmholtz electromagnetic coil integrated with a ultrasound imaging modality. (B) The ultrasound transducer.

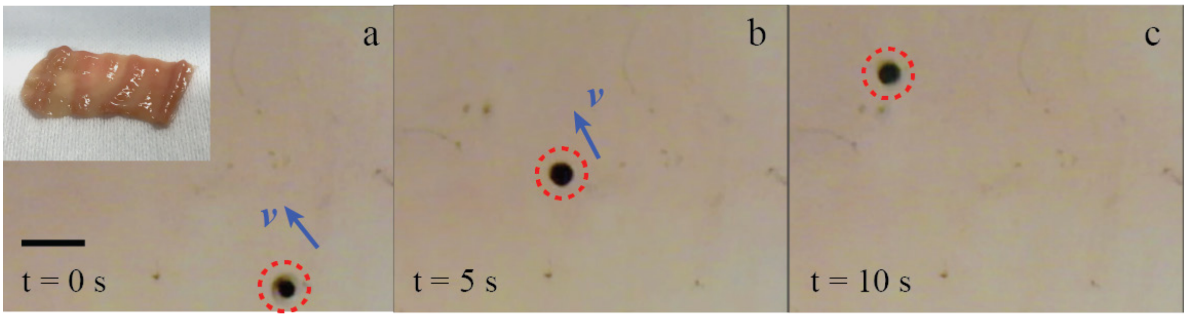

Supplementary Figure 16: The actuation of a vortex-like swarm on an intestinal tract sample. The red dotted circles indicate the location of the swarms. The blue arrows represent the real-time direction of the velocity of the swarms. The scale bar indicates 2 mm.

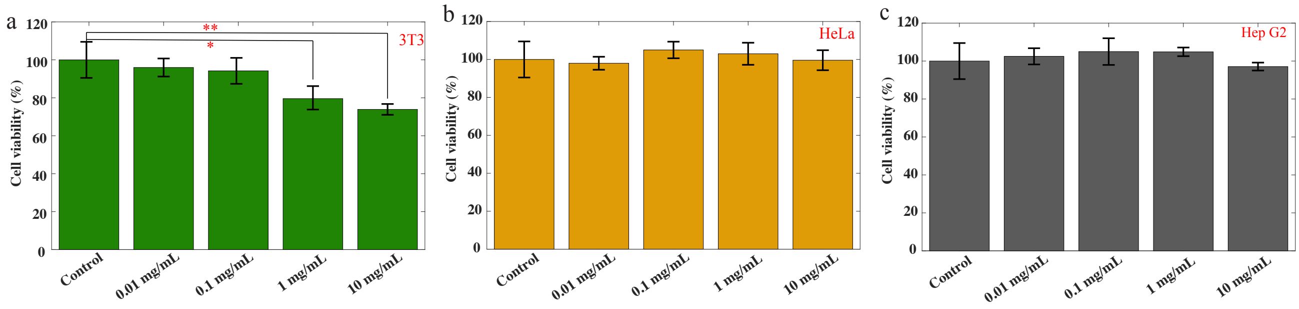

Supplementary Figure 17: Cytotoxicity test. Cell viability studies of the 3T3, HeLa, and HepG2 cells after being cocultured with nanoparticles with a wide range of concentrations, i.e., 0.01, 0.1, 1, and 10 mg mL^{-1}, after 24 h of incubation. The control group proceeded without any nanoparticles. The error bars were obtained from 3 to 5 groups of experiments. Asterisks denote the level of significance: * for $p < 0.05$, and ** for $p < 0.01$.

Supplementary Tables

Parameter	Value	Details
ξ (1/s)	15	Vorticity of each vortex (Supplementary Fig. 1b)
d (μm)	200	Distance between two vortices (Supplementary Fig. 1b)
r_v (μm)	50	Radius of each vortex (Supplementary Fig. 1b)
r_p (μm)	1	Radius of each particle (Supplementary Fig. 1f)
μ_p [V· s/ (A· m)]	80×10^{-6}	Magnetic permeability of nanoparticles (Supplementary Fig. 1f)
μ_r [V· s/ (A· m)]	1.257×10^{-6}	Magnetic permeability of air (Supplementary Fig. 1f)
B_0 (mT)	10	Applied external magnetic field strength (Supplementary Fig. 1f)

Supplementary Table 1: The parameters used in the simulation in Supplementary Figure 1b and f.

Supplementary Notes

Supplementary Note 1: Swarming methodology and mechanisms

The schematic generation process of a fluidic-induced swarm is shown in Supplementary Figure 1a. Dispersed paramagnetic nanoparticles tend to attract each other to form chain-like structures in a magnetic field (Supplementary Figure 1a I - II). Hereby, we regard nanoparticle chains as the minimum units in the analysis. A particle chain actuated by a rotating magnetic field in fluid will generate a local fluidic vortex, as shown by the blue arrows in Figure 1a. The vorticity $\vec{\xi}$ of a flow field with velocity distribution $\vec{u}$ is defined by:

$$\vec{\xi} = \nabla \times \vec{u} = \left(\frac{\partial u_z}{\partial y} - \frac{\partial u_y}{\partial z}, \frac{\partial u_x}{\partial z} - \frac{\partial u_z}{\partial x}, \frac{\partial u_y}{\partial x} - \frac{\partial u_x}{\partial y}\right), \quad (1)$$

where $\mathbf{u_x}$, $\mathbf{u_y}$, and $\mathbf{u}_z$ are the three components of $\vec{\mathbf{u}}$ along the three axes of Cartesian coordinate. In a two-dimensional vortex (in x-y plane), because the flow is confined in the plane, only the z-component of the vorticity is non-zero, Supplementary Equation 1 can be simplified to:

$$\vec{\boldsymbol{\xi}} = \left(\frac{\partial \mathbf{v_y}}{\partial x} - \frac{\partial \mathbf{v_x}}{\partial y}\right)\vec{\mathbf{z}}. \quad (2)$$

The merging of vortices induced by rotating particle chains is the main reason for the generation of a fluidic-induced swarm, as shown in Supplementary Figure 1a III - V. The induced fluidic vortices exerts long-range attractive interaction forces on adjacent particle chains, which gradually reduces the distances among them. Finally, when the distance of two rotating chains is close enough, the vortices induced by them will merge. The simulation results of the vortex merging are presented in Supplementary Figure 1b. Initially, two identical vortices are distributed, and driven by the attractive fluidic interaction induced by the advection of vorticity, the vortices move towards each other. When they come into contact, they rapidly deformed into prolate shapes. Then, an elliptical shape with two filaments of vorticity is formed. Finally, a circular vortex pattern is generated, while the filaments gradually roll-up around the core of the vortex and dissipated. As a result, the chains keep self-rotating following the rotating magnetic field and, meanwhile, they begin to perform coaxial rotation due to the fluidic interaction. After continuing merging processes, the initial great numbers of vortices will merge into one vortex or a few major vortices induced by multiple particle chains, and the fluidic-inducedd swarm is formed. The simulation results validate the merging process of vortices, and therefore, can support the hypothesis that this kind of swarm is generated mainly based on fluidic interactions. The experimental results of the generation of a fluidic-induced swarm is demonstrated in Supplementary Figure 1c. The nanoparticles are dispersed uniformly at the initial state. After a rotating magnetic field is applied, the nanoparticle chains are observed to perform self-rotation at first, then a relatively high concentrated region of nanoparticles is formed, which is the prototype of a fluidic-induced swarm. Finally, most of the particle chains are gathered into the core of the vortex, and a dynamic-equilibrium fluidic-induced swarm is formed with the particle chains rotating synchronically.

The schematic process of the generation of an MF-induced swarm is shown in Supplementary Fig. 1D. We take the initial situation that dispersed nanoparticles form two long particle chains as an example. When the oscillating magnetic field (Figure 1b) is applied, the long chains are firstly broken into several shorter pieces at stage III. Because at this moment, the magnetic field strength is low, and the chain-chain magnetic interactions are not sufficient to actuate the chains. Therefore, the relative locomotion of the chains are maintained. After the field strength reaches the maximal value (stage IV), the interaction among particle chains become stronger, which makes their distribution along x-axis narrower and y-axis longer. Dipole interactions are induced between two paramagnetic particle chains in a magnetic field, and the simulation results are presented in Supplementary Figure 1f. The particle chains will induce local magnetic fields, which influences the external magnetic field. The white arrows indicate the directions of superposed magnetic field, and the colour map shows the field strength that is induced by the particle chains. The simulation results of the induced forces exerted on the chains are shown by the green arrows in Supplementary Figure 1f. The dipole forces tend

to re-arrange the particle chains into a long (along y-axis) and narrow (along x-axis) pattern. As a result, the ribbon-like swarm is formed mainly due to the dipole-dipole interactions among the particle chains.

The experimental results of MF-induced swarm are shown in Supplementary Figure 1d. A three-axial Helmholtz electromagnetic coil setup is applied for magnetic actuation, as shown in Supplementary Figure 1e. The control signal is programmed on the control PC, and the experimental results are observed using the CCD camera. Actuated by the oscillating magnetic fields, the nanoparticles locally gathered into chain-like dynamic patterns. After a series of self-merging processes of subswarms, a ribbon-like swarm is generated, which is mainly resulted from dipole-dipole interactions among magnetic agents. The SEM image of the MF-induced swarm is shown in the inset of Supplementary Figure 1d. The key parameters used in the simulations are listed in Supplementary Table 1.

Supplementary Note 2: Reconfiguration of swarms in fluids with multiple ionic strengths and viscosities

The relationship between amplitude ratio γ of the applied magnetic field and the aspect ratio α of the ribbon-like swarm (MF-induced) in fluids with different ionic strengths is demonstrated in Supplementary Figure 4. With the same amplitude ratio, the ribbon-like swarm tends to have lower aspect ratio if the ionic strength of the fluids is higher. Meanwhile, the swarm begin to form at γ=3 in 0.2×, 0.4×, 0.6× PBS, and when the ionic strength continues to increase, the lowest amplitude ratio γ_l for triggering the generation of the swarm becomes larger, e.g. γ_l =4, 5, 6 in 0.8×, 1×, 2× PBS, respectively. A high ionic strength enhances particle-particle attractive interactions, and with the same oscillating frequency, the particle chains in fluids with more ions will be longer and bulkier. Therefore, the reconfiguration process of the MF-induced swarms may be hindered, and the chains cannot be broken and re-assembled sufficiently, which makes the aspect ratio smaller. The reason also explains the curves in Supplementary Figure 5. With a higher viscosity, the aspect ratio of the MF-induced swarm will be larger, because particle chains tend to form shorter ones, which makes the reconfiguration process more sufficient. However, because the generation of MF-induced swarm requires fast response of magnetic particle chains in fluids, if the viscosity continues to increase, which prevents the agents from fast reconfiguration, the MF-induced swarm may not be able to form.

Supplementary Note 3: Generation conditions for swarms in bio-fluids.

The generation conditions for the MF-induced swarms in FBS are demonstrated in Supplementary Figure 8. In FBS, when amplitude ratio γ is low (blue crosses), particles will form massive zig-zag patterns. In the region with green circles, straight patterns which uncontrollably elongate are formed. With the increase of γ (blue diamonds), the particles generate unstable ribbon-like swarms with continuous pattern reconfiguration. A dynamic-stable ribbon-like swarm is formed in the region of red asterisks, which is highlighted by the red area. Multiple long chain-like patterns will be formed if γ becomes even larger (black crosses).

The generation condition for the medium-induced swarms in HA is demonstrated in Supplementary Figure 9. When the applied magnetic field strength is not sufficiently strong to actuate the nanoparticle chains, the cases

are labeled using blue "×". The medium-induced swarms with a low percentage of central area and loose cores are presented by the green circle. The red area surrounded by the red asterisks indicates the proper conditions for the generation of the medium-induced swarms.

Supplementary Note 4: Reconfiguration of MF-induced swarms in bio-fluids

By tuning input amplitude ratio, the aspect ratio of the MF-induced swarms will change accordingly, and the results are shown in Supplementary Figure 10. In all four bio-fluids, the swarms have larger aspect ratios when the input frequency is higher, and the largest aspect ratio reaches approximately 38, when the swarm is actuated by 30 Hz-frequency fields in FBS. Meanwhile, in DI water, the differences on aspect ratios between cases is larger than those in gastric acid. The average length of nanoparticle chains is a major factor for the swarm reconfiguration, and in DI water, when the amplitude ratio and field strength is fixed, the chain length is only determined by the frequency. In gastric acid, the additional ions in fluids induce attractive electrostatic forces among nanoparticles, which potentially reduces the difference on chain length with different input frequencies. Therefore, the differences on aspect ratios are smaller in fluids with high ionic strengths.

Supplementary Note 5: Navigated locomotion of an MF-induced swarm in 4× diluted blood.

In this part, we demonstrate the feasibility of making navigated locomotion of MF-induced swarms in 4× diluted blood, as shown in Supplementary Figure 11. The red dashed arrow indicates the moving trajectory of the swarm, and the white arrows schematically indicate the flow field around the swarm.

Supplementary Note 6: Investigations of MF-induced swarms formed by corona-coated nanoparticles.

The incubation steps are briefly described as follows: The in vitro protein corona was allowed to form by adding magnetite nanoparticles (6.6 mg/mL) into human plasma at a ratio of 1:4 and incubating for 10 minutes at room temperature by gentle shaking. According to the literature (Hadjidemetriou et. al., Time-evolution of in vivo protein corona onto blood-circulating PEGylated liposomal doxorubicin (DOXIL) nanoparticles, Nanoscale, 2016), protein corona formation occurs within 1 minute and therefore 10 minutes of ex vivo incubation were chosen in this case to ensure a well-established and complete protein corona formation onto the surface of the nanoparticles. After the incubation, the nanoparticles were separated from plasma using a magnet and were thoroughly washed by adding HEPES buffer to remove any unbounded or weakly bounded proteins. The recovered corona-coated nanoparticles were then used for the magnetic actuation. From the results, it can be observed that, despite the influence from the corona and extra charges, the magnetic interaction among the nanoparticles is still dominating. The generation, reconfiguration and navigated locomotion processes of an MF-induced swarm are realized successfully.

Supplementary Note 7: Investigation of the influence of dilution effect in vitreous humor on swarms.

Hereby, we first investigate the effects of partial dilution, which may be cause by the injected particle suspension locally, and the experimental results are shown in Supplementary Figure 13. We first suspend nanoparticles in blue dye, and inject the blue nanoparticle suspension into the vitreous humor. The region of dye indicates the location that may be diluted by the injected solution. After the rotating magnetic field is applied, the spread nanoparticles immediately gathered into a circular swarm, and it can move effectively in the region of dye, as shown in Supplementary Figure 13b and c. In Supplementary Figure 13d-f, the swarm begin to move out of the region of dye, where the effect of dilution can be neglected. The swarm can still make locomotion efficiently. Based on the results, the locomotion capability of the swarm is not dependent on the partial dilution of the vitreous humor due to the injected suspension.

It is also noted that, due to the high viscosity and dense meshes of vitreous humor, the natural diffusion of the blue dye is significantly limited. The major change of the contour of the dyed region is highlighted by the yellow dashed arrows in Supplementary Figure 13d, which is caused by the fluid interaction induced by the swarm. Due to the rotation of the swarm (indicated by the red arrows), the dye at the location pointed by the upper arrow is pushed back a bit to the left region, while the dye at the lower arrow is pulled to the right region with the swarm.

Moreover, we have also excluded the possibility that the locomotion of the swarm is mainly due to the overall dilution of the vitreous humor. We characterize the viscoelasticity of the fresh vitreous humor and the mixed sample of vitreous humor and PBS solution. In the ex-vivo experiments (in bovine eyeballs), 200 μL PBS solution with suspended nanoparticles is injected into the eyeball (~ 5 mL vitreous humor). The proportion of vitreous and PBS solution in the characterization is kept the same with that in the ex-vivo experiments. The results are shown in Supplementary Figure 14. It can be observed that, the viscoelasticity of the two samples are maintained in the same range. The small deviation of the results may be caused by the measuring error of the equipment. As a result, the injection of the particle suspension will not significantly change the viscoelasticity of the vitreous humor, which is not the main reason for the locomotion of the swarm neither.

We also characterize the physical properties of vitreous humor before and after the magnetic actuation experiments. The results of viscoelasticity are shown in Supplementary Figure 14. The viscoelasticities of original sample and the sample after the actuation experiments (a swarm move in it for ~ 2 min) are compared. It is observed that, the viscoelasticities of these two samples are in the same range. As a result, the locomotion of the swarm will not influence the physical properties of the vitreous humor.

Supplementary Note 8: Actuation of a swarm on a mucosa sample.

We use a piece of porcine intestinal tract as the substrate, and conduct swarm actuation experiments on it. It is noted that, the original mucus layer is remained on the intestinal sample. In our first trial, we directly drop the nanoparticle suspension onto the sample, but when the nanoparticles contact the surface of the intestinal tract,

they cannot be actuated again due to the overwhelming sticking surface force. In order to solve this issue, we conduct the following steps.

We first collect some mucus into an open tank with a silica substrate, and 4 μL nanoparticle suspension with a concentration of 6 mg/mL is injected into the mucus. Then a rotating magnetic field is applied, and because mucus is full of mesh-like structures, spread nanoparticles immediately gather into a swarm tangling with the fibres. In this case, the swarm is an ensemble with a relatively stable structure, and meanwhile, the magnetic torque and force induced are significantly enlarged. The swarm is subsequently retracted from the mucus, and injected onto the intestinal sample with a mucus layer. The experimental results is shown in Supplementary Figure 16. After applying a rotating magnetic field with a pitch angle of 5°, the swarm can be actuated and navigated efficiently due to the large actuation force. As a conclusion, the swarm can also be actuated on a mucosa sample.

Supplementary Note 9: Cytotoxicity tests.

The cytotoxicity of the nanoparticles used for the swarm are evaluated by the 3-(4, 5-dimethylthiazol-2-yl)-5-(3-carboxymethoxyphenyl)-2-(4-sulfophenyl)-2H-tetrazolium (MTS) cell viability assay, which is a colorimetric method that determines the cell viability of both normal cells (3T3 cells) and tumor cells (HeLa cells and HepG2 cells) by detecting the brownish formazan product in the cells produced by the metabolic activity of cells. Cell viabilities with different nanoparticle concentrations are tested, i.e., 0.01, 0.1, 1, and 10 mg/ mL. Cell viability of less than 70% denotes cytotoxicity of the applied materials according to ISO 10993-5. The results are shown in Supplementary Figure 17. For the 3T3 cells (Supplementary Figure 17a), even though the viability trend shows a slight decrease with the increase in the nanoparticle concentrations, the cell viability is still maintained at a high level (>70%) when the FMP concentration is as high as 10 mg/mL after 24 h of incubation, which denotes a minor cytotoxicity of the nanoparticles to normal cells. As for the HeLa cells (Supplementary Figure 17b) and HepG2 cells (Supplementary Figure 17c), the cell viability is ≈100% and remain unchanged with the increase in the nanoparticle concentration from 0.01 mg/mL to the high concentration of 10 mg/mL (Supplementary Figure 17b, c). The results indicated that the nanoparticles exhibited very low cytotoxicity to the tumor cells in vitro at both low and high concentrations, and hence, the results testify the feasibility of applying a swarm for targeted delivery.
