# Supplementary material for: Active generation and magnetic actuation of microrobotic swarms in bio-fluids
Source: Nat Commun. 2019 Dec 10;10:5631. doi: 10.1038/s41467-019-13576-6 (PMC6904566; doi:10.1038/s41467-019-13576-6)
Supplement: Supplementary file 2 — Description of Additional Supplementary Files [file 41467_2019_13576_MOESM2_ESM.pdf]

Description of Additional Supplementary Files

File Name: Supplementary Movie 1
Description: Generation of magnetic field-induced and medium-induced swarms in various bio-fluids.

File Name: Supplementary Movie 2
Description: Locomotion of swarms made in four different bio-fluids.

File Name: Supplementary Movie 3
Description: Generation and navigated locomotion of magnetic field-induced swarms in 4× diluted blood.

File Name: Supplementary Movie 4
Description: Navigated locomotion of a medium-induced swarm in vitreous humor.

File Name: Supplementary Movie 5
Description: Generation and navigated locomotion of a medium-induced swarm in vitreous humor with ultrasound feedback.

File Name: Supplementary Movie 6
Description: Generation of a medium-induced swarm in a bovine eyeball.

File Name: Supplementary Movie 7
Description: Navigated locomotion of a large medium-induced swarm (r~2.2 mm) made in a bovine eyeball.

File Name: Supplementary Movie 8
Description: Navigated locomotion of a small medium-induced swarm (r~0.5 mm) made in a bovine eyeball.

File Name: Supplementary Movie 9
Description: Navigated locomotion of a small medium-induced swarm (r~0.7 mm) made in a bovine eyeball.
